# Blood CXCR3^+^ CD4 T Cells Are Enriched in Inducible Replication Competent HIV in Aviremic Antiretroviral Therapy-Treated Individuals

**DOI:** 10.3389/fimmu.2018.00144

**Published:** 2018-02-05

**Authors:** Riddhima Banga, Francesco A. Procopio, Alessandra Ruggiero, Alessandra Noto, Khalid Ohmiti, Matthias Cavassini, Jean-Marc Corpataux, William A. Paxton, Georgios Pollakis, Matthieu Perreau

**Affiliations:** ^1^Service of Immunology and Allergy, Lausanne University Hospital, University of Lausanne, Lausanne, Switzerland; ^2^Department of Clinical Infection, Microbiology and Immunology (CIMI), Institute of Infection and Global Health (IGH), University of Liverpool, Liverpool, United Kingdom; ^3^Infectious Diseases, Lausanne University Hospital, University of Lausanne, Lausanne, Switzerland; ^4^Vascular Surgery, Lausanne University Hospital, University of Lausanne, Lausanne, Switzerland

**Keywords:** T follicular helper cells, replication competent virus, circulating T follicular helper cell counterpart, CXCR3, lymph node

## Abstract

We recently demonstrated that lymph nodes (LNs) PD-1^+^/T follicular helper (Tfh) cells from antiretroviral therapy (ART)-treated HIV-infected individuals were enriched in cells containing replication competent virus. However, the distribution of cells containing inducible replication competent virus has been only partially elucidated in blood memory CD4 T-cell populations including the Tfh cell counterpart circulating in blood (cTfh). In this context, we have investigated the distribution of (1) total HIV-infected cells and (2) cells containing replication competent and infectious virus within various blood and LN memory CD4 T-cell populations of conventional antiretroviral therapy (cART)-treated HIV-infected individuals. In the present study, we show that blood CXCR3-expressing memory CD4 T cells are enriched in cells containing inducible replication competent virus and contributed the most to the total pool of cells containing replication competent and infectious virus in blood. Interestingly, subsequent proviral sequence analysis did not indicate virus compartmentalization between blood and LN CD4 T-cell populations, suggesting dynamic interchanges between the two compartments. We then investigated whether the composition of blood HIV reservoir may reflect the polarization of LN CD4 T cells at the time of reservoir seeding and showed that LN PD-1^+^ CD4 T cells of viremic untreated HIV-infected individuals expressed significantly higher levels of CXCR3 as compared to CCR4 and/or CCR6, suggesting that blood CXCR3-expressing CD4 T cells may originate from LN PD-1^+^ CD4 T cells. Taken together, these results indicate that blood CXCR3-expressing CD4 T cells represent the major blood compartment containing inducible replication competent virus in treated aviremic HIV-infected individuals.

## Introduction

Antiretroviral therapy (ART)-treated HIV-infected individuals interrupting treatment experience HIV viremia rebound within 2–3 weeks (1, 2), demonstrating that HIV persists despite therapy and that conventional antiretroviral therapy (cART) does not cure HIV in its actual setting. One of the main mechanisms by which HIV persists is attributed to the ability of the virus to infect activated CD4 T cells entering a quiescent state (3), thereby establishing a latent HIV cell reservoir (4). Latently HIV-infected CD4 T cells are transcriptionally silent and are therefore not targeted by cART or by the immune system (5). As a direct consequence, infected CD4 T cells containing HIV DNA are detectable in virtually all ART-treated HIV-infected individuals (6). Since, ART does not target HIV-infected cells, it has been estimated, based on the half life of memory CD4 T cells, that as long as 70 years of ART might be required for the eradication of the latent reservoir (4). Moreover, mechanisms such as homeostatic proliferation may also contribute to further increase the stability of the latent HIV reservoir (3, 4, 7, 8).

The composition of the latent HIV reservoir is complex and different cell subsets may contribute such as CD4 T cells, monocytes and macrophages (8–10). Among the CD4 T-cell subsets, central memory (CM; defined by CD45RA^–^CCR7^+^CD27^+^) and transitional memory (TM; CD45RA^–^CCR7^–^CD27^+^) CD4 T cells were identified as major CD4 T-cell populations contributing the most to the latent HIV-1 reservoir in blood (7). Additional CD4 T-cell populations enriched in cells containing replication competent viruses were later described such as memory CD4 T cells with stem-cell like properties (11). On the same line, Descours et al. identified CD32a (low-affinity receptor for the immunoglobulin G Fc fragment) as a specific marker of latently HIV-infected quiescent CD4 T cells (12). However, lymphocytes reside predominantly within lymphoid organs while blood contains only 2% of the total body’s lymphocytes. More importantly, lymphocyte populations within the tissues are phenotypically and functionally distinct from those in blood (13). The recently described T follicular helper (Tfh) cells illustrate this difference (14, 15). In this regard, lymph nodes (LN) memory Tfh cells and to a lesser extent memory CXCR5^−^PD-1^+^ CD4 T cells were previously shown to serve as the major CD4 T-cell compartments for HIV replication, production and infection in viremic HIV-1-infected individuals (16). In addition, we have recently demonstrated that LN PD-1^+^/Tfh CD4 T cells isolated from ART-treated aviremic HIV-infected individuals were enriched in cells containing replication competent and infectious HIV as compared to any other PD-1 negative memory CD4 T-cell populations isolated from blood or LN (17). This phenomenon is probably associated with the limited cytotoxic CD8 T-cell access to germinal centers (GCs) (18, 19) where infected Tfh cells locate. However, since HIV-infected effector Tfh cells surviving viral cytopathic effects and escaping extra-follicular CD8 T-cell mediated clearance may convert to memory CD4 T cells and recirculate in blood (20, 21), we hypothesized that blood circulating Tfh cell counterpart (circulating Tfh or cTfh) may therefore be enriched in cells containing replication competent virus.

Circulating Tfh (cTfh) cells were initially defined by the expression of CXCR5 and showed enhanced capacity to provide naïve and memory B-cell help (22). Later, cTfh cells were subdivided into various subsets based on the expression of CXCR3, CCR4, CCR6, PD-1, and ICOS (23–25). Indeed, T-cell migration is orchestrated by the combination of cell-adhesion molecule and chemokine receptor expression (20) Notably, CXCR5 expression allows cell migration to B cell follicles, CXCR3 expression allows cell migration to inflamed tissues and CCR4 and CCR6 expression allows migration to skin and mucosal tissues, respectively (26, 27).

In this context, we have investigated the distribution of replication competent and infectious virus within different blood memory CD4 T-cell populations identified on the basis of their migratory potential determined by the chemokine receptor expression and compared to memory CD4 T-cell populations isolated from LNs of cART-treated HIV-infected individuals. Briefly, blood circulating memory (CD45RA^−^) CD4 T-cell populations were identified on the basis of expression of CXCR3, CXCR5, CCR4, and CCR6, i.e., CXCR3^+^CXCR5^−^ (CXCR3^+^), CCR4^+^CCR6^−^ (CCR4^+^), CCR4^+^CCR6^+^, CXCR3^−^CXCR5^+^, and CXCR3^+^CXCR5^+^ CD4 T cells, the latter two populations corresponding to “cTfh” (22, 23) and “Th1-like cTfh” (28). On the other hand, LN memory (CD45RA^−^) CD4 T-cell populations were identified on the basis of the expression of CXCR5 and/or PD-1 expression as previously described (17).

In the present study, we show that blood CXCR3-expressing but not CXCR5-expressing memory CD4 T-cell subset was significantly enriched in cells containing inducible replication competent virus and contributed the maximum to the total pool of cells containing replication competent and infectious virus in blood. However, the enrichment of blood CXCR3-expressing memory CD4 T cells with cells containing replication competent virus was not associated with increased level of activation, HIV coreceptor expression or reduced HIV restriction factor expression, nor with an enrichment in cells harboring central or transitional memory phenotype.

To determine whether blood CXCR3-expressing memory CD4 T cells containing replication competent virus may originate from LN Tfh cells, proviral EnvV1-V4 sequences of blood and LN CD4 T-cell populations of ART-treated aviremic HIV-infected individuals were analyzed but did not indicate virus compartmentalization. We then investigated whether the composition of blood HIV reservoir may reflect the polarization of LN CD4 T cells at the time of reservoir seeding and showed that LN PD-1^+^/Tfh cells of viremic untreated HIV-infected individuals expressed significantly higher levels of CXCR3 as compared to CCR4 and/or CCR6 which is consistent with previous studies performed in chronically SIV-infected macaques (29).

Taken together, these results indicate that blood CXCR3-expressing CD4 T cells represent the major blood compartment containing inducible replication competent virus in treated aviremic HIV-infected individuals. However, additional studies would be needed to determine their potential origins and the mechanism by which HIV-infected cells accumulated within this particular subset.

## Materials and Methods

### Ethics Statement

The present study was approved by the Institutional Review Board of the Centre Hospitalier Universitaire Vaudois, and all subjects gave written informed consent.

### Study Group and Cell Isolation

Nine viremic untreated HIV-1-infected adult volunteers and 19 aviremic ART-treated HIV-infected individuals were enrolled in the present study (Table 1). No predetermined statistical analysis was performed for sample size and was estimated based on a previously published study (17). As inclusion criteria only patients under ART for atleast 12 months with undetectable HIV-1 viremia (<20 HIV-1 RNA copies/mL) were enrolled. As exclusion criteria, individuals experiencing blips of viremia (>50 HIV-1 RNA copies/mL of plasma) within the last 12 months were not enrolled. Leukapheresis and blood samples were obtained at the local blood bank (Centre de transfusion sanguine (CTS), Lausanne, Switzerland). Blood mononuclear cells were isolated as previously described (30).

**Table 1:** Characteristics of study group.

Subject ID	Duration of HIV infection (years)	CD4 count (cells/μl)^a^	Viral load (copies/mL)^a^	Time on HAART (years)	HAART regimen	Assays performed in blood	Assays performed in LN
#10	9.1	543	<20	8.8	EFV, 3TC	INT DNA, VOA, FC	NA
#12	8.4	453	<20	2.9	3TC/r, ABC	INT DNA, VOA, FC	NA
#14	10.1	1,691	<20	3.5	EFV, TDF, FTC	INT DNA, VOA, FC	INT DNA, VOA
#16	25.9	487	<20	2	ETR; DRV/r; RAL	INT DNA, VOA, FC	NA
#18	2.6	376	<20	2.5	FTC, TDF, EFV	INT DNA, VOA, FC	NA
#20	25.3	480	<20	14.1	ETR, DRV/r, RAL	INT DNA, VOA, FC	NA
#26	9.6	666	<20	2	3TC, ABC, DTG	INT DNA, VOA, FC	INT DNA, VOA
#35	2.1	1,219	<20	1.8	FTC, TDF, EVG	INT DNA, SEQ, VOA, FC	INT DNA, SEQ, VOA
#43	2.8	605	<20	2.8	3TC, ABC, DTG	VOA, FC	NA
#45	8	442	<20	7.9	FTC, TDF, ETR	INT DNA, VOA, FC	NA
#75	4.7	417	<20	3.2	FTC, TDF, EFV	INT DNA, VOA	VOA
#94	7	440	<20	6.9	FTC, TDF, EVG	INT DNA, SEQ, VOA	VOA, SEQ
#107	1.9	598	<20	1.8	FTC, TDF, EFV	INT DNA, VOA	VOA
#077	6.6	560	<20	6	3TC, ABC, DTG	SEQ	SEQ
#24	10.7	629	<20	3.9	FTC, TDF, EFV	NA	INT DNA, VOA
#11	16.2	811	<20	11.7	FTC, TDF, ATV/r	NA	INT DNA, VOA
#04	2.9	424	<20	1.8	EFV, TDF, FTC	NA	INT DNA, VOA
#25	28.5	439	<20	4.6	FTC, TDF, RAL	NA	INT DNA, VOA
#42	11.1	949	<20	6.6	FTC, TDF, ATV/r	NA	INT DNA, VOA
#106	0.1	501	160,000	0	NA	NA	MC
#140	0.08	427	360,000	0	NA	NA	MC
#119	1.3	498	13,000	0	NA	NA	MC
#124	0.06	468	510,000	0	NA	NA	MC
#113	31.98	594	640	0	NA	NA	MC
#117	5.42	504	54,000	0	NA	NA	MC
#125	0.16	511	17,000	0	NA	NA	MC
#118	0.06	538	14,000	0	NA	NA	MC
#SA150	10	578	6,900	0	NA	NA	MC

*^a^CD4 cell count and viral load assessments are at the time of enrollment in the study*.

*ETR, Etravirine; FTC, Emtricitabine; TDF, Tenofovir disoproxil fumarate; ATV/r, Atazanavir boosted with ritonavir; 3TC, Lamivudine; ABC, Abacavir; DRV/r, Darunavir boosted with ritonavir; EFV, Efavirenz; RAL, Raltegravir; DTG, Dolutegravir; EVG, Elvitegravir; LN, lymph node; INT DNA, integrated DNA; VOA, viral outgrowth assay; FC, flow cytometry; SEQ, sequencing; MC, Mass cytometry; NA, not applicable*.

### Reagents and Cell Culture

Cells were cultured in RPMI (Gibco; Life Technologies) containing 10% heat-inactivated fetal bovine serum (FBS; Institut de Biotechnologies Jacques Boy), 100 IU/mL penicillin, and 100 µg/mL streptomycin (Bio Concept).

### Antibodies

The following antibodies were used: APC-H7-conjugated anti-CD3 (clone SK7), PB or FITC or PE-CF594-conjugated anti-CD4, PerCP-Cy5.5-conjugated anti-CD8 (clone SK1), (clone RPA-T4), (2G8), PE-conjugated anti-CCR6 (11A9), PE-Cy7-conjugated anti-CCR4 (1G1), V450-conjugated anti-HLA-DR (clone G46-6), PE-Cy7-conjugated anti-CD25 (clone M-A251), PeCy5-conjugated anti-CXCR4 (12G5), AlexaFluor700-conjugated anti-CCR5 (HEK/1/85a), PB or PeCy7 conjugated anti-PD-1 (EH12.1), anti-CCR7 (2H4), AlexaFluor700-conjugated anti-CD27 (M-T271), PE-conjugated anti-Ki67 (B56) purified coating anti-CD3 (clone UCHT1) and anti-CD28 (clone CD28.2) mAbs were purchased from BD (Becton Dickinson, San Diego CA, USA). PB-conjugated anti-CXCR3 (1C6) and PE or APC or PerCP-eFluor710-conjugated anti-CXCR5 mAbs were all purchased from Biolegend (Switzerland). ECD-conjugated anti-CD45RA (clone 2H4) was purchased from Beckman Coulter (Brea CA, USA) and anti-SAMHD1 (611-625) was purchased from Thermo Scientific (Switzerland).

### Sorting of Blood and LN Memory CD4 T-Cell Populations

Sorting of chemokine-receptor-expressing memory CD4 T cells was performed using FACS Aria as previously described (17). Briefly, cryopreserved blood mononuclear cells were thawed and CD4 T cells were enriched using EasySep Human CD4 T-cell enrichment kit (StemCell Technologies, Cambridge MA, USA). CD4 T cells were then stained with Aqua LIVE/DEAD stain kit (4°C; 15 min) and then to simultaneously investigate the expression of chemokine receptors on CD4 T cell membrane, with anti-CD3 APCH7, anti-CD4 FITC, anti-CD45RA ECD, anti-CXCR3 PB, anti-CXCR5 APC, anti-CCR4 PE-Cy7 and anti-CCR6 PE (4°C; 25 min). Viable CD4 memory (CD45RA^−^) CXCR3^+^CXCR5^−^, CXCR3^−^CXCR5^+^, CXCR3^+^CXCR5^+^, CXCR3^−^CXCR5^−^CCR4^+^CCR6^−^, and CXCR3^−^CXCR5^−^CCR4^+^CCR6^+^ T-cell populations were then sorted. In parallel, cryopreserved LN mononuclear cells were also thawed and stained with Aqua LIVE/DEAD stain kit (4°C; 15 min) and then with anti-CD3 APCH7, anti-CD4 FITC, anti-CD45RA ECD, anti-PD-1 PE-Cy7, and anti-CXCR5 APC (at 4°C; 25 min) and viable CD4 memory (CD45RA^−^) CXCR5^−^PD-1^−^ (DN), CXCR5^+^PD-1^−^ (single CXCR5), and total PD-1^+^ cells were sorted. The grade of purity of the sorted cell populations was >97% in all sorting experiments.

### Integrated HIV-1 DNA Quantification

Blood and LN memory CD4 T cells of ART-treated HIV-infected individuals were sorted and CD3 gene copy numbers were determined as previously described (31). The frequency of HIV-1 integrated DNA per million of cells was then calculated as previously described (31).

### Viral Outgrowth Assay

Multiple cell concentrations, i.e., fivefold limiting dilutions: 5 × 10^5^, 10^5^, 2 × 10^4^, and 4 × 10^3^ for blood CD4 T cell populations and 10^5^, 2 × 10^4^, and 4 × 10^3^ for LN CD4 T-cell populations of sorted viable blood and LN memory CD4 T cells isolated from ART-treated HIV-infected individuals were cultured with allogenic fresh CD8-depleted blood mononuclear cells (10^6^ cells/mL) from HIV-uninfected individuals in the presence of anti-CD3/anti-CD28 MAb coated plates (10 µg/mL) for 3 days. Cells were then carefully transferred to new uncoated plates post 3 full days of activation. All conditions were cultured in complete RPMI supplemented with IL-2 (50 units/mL) for 14 days. Medium was replenished at day 5, and re-supplemented with cytokines. Supernatants were collected at day 14. The presence of HIV-1 RNA was assessed by COBAS^®^ AmpliPrep/TaqMan^®^ HIV-1 Test (Roche; Switzerland). Wells with detectable HIV-1 RNA (≥20 HIV-1 RNA copies/mL) were referred to as HIV-1 RNA-positive wells. RUPM frequencies (32) were estimated by conventional limiting dilution methods using Extreme Limiting Dilution analysis (http://bioinf.wehi.edu.au/software/elda/) (33). The estimation of each population’s contribution to the overall pool of HIV-infected cells within blood compartment or within LN compartment was performed as previously described (17) (Table S1 in Supplementary Material). Briefly, estimated contribution of memory CD4 T cell population A from blood or LN to the pool of cells containing replication competent virus in blood or LN compartments = [(% of memory CD4 T cell population A from compartment X among total memory CD4 T cells from compartment X) × (estimated RUPM freq. of memory CD4 T cell population A from compartment X)] × [% of the estimated number of memory CD4 T cells present in compartment A among the sum of the memory CD4 T cells present in blood or LN]/[(sum of absolute values of cells containing replication competent virus within each memory CD4 T cell population) × 100]. In addition, estimated contribution of memory CD4 T cell population A from compartment X to the pool of cells containing replication competent virus in both blood and LN compartments = [(% of memory CD4 T cell population A from compartment X among total memory CD4 T cells from compartment X) × (estimated RUPM freq. of memory CD4 T cell population A from compartment X)] × [% of the estimated number of memory CD4 T cells present in compartment A among the sum of the memory CD4 T cells present in both blood and LN]/[(sum of absolute values of cells containing replication competent virus within each memory CD4 T cell population from compartment X and Y) × 100]. The estimated number of total blood and LN memory CD4 T cells was obtained from Ganusov et al. (34).

### *In Vitro* HIV-1 Infection Assay

The *in vitro* infection assay was performed as previously described (17). Briefly, preactivated CD8-depleted blood mononuclear cells isolated from HIV-uninfected individuals were washed and exposed for 6 h at 37°C to 100 µl of VOA supernatants (obtained from the highest concentration, i.e., 5 × 10^5^) collected at day 14 from all chemokine receptor expressing blood memory CD4 T-cell populations in VOA. Following 6 h exposure, cells were washed twice with complete medium and cultured for 14 additional days in complete RPMI medium. The presence of infectious HIV-1 particles was determined in culture supernatants at day 0 and 14 post inoculations as assessed by HIV-1 RNA assay (COBAS^®^ AmpliPrep/TaqMan^®^ HIV-1 Test) as previously described (17). For this assessment of HIV RNA, all samples were prediluted 1/10 in basematrix buffer (RUWAG Handels AG).

### Proviral Sequencing of Env V1-V4 Region

Proviral sequencing of Env V1-V4 was performed on three aviremic ART-treated HIV-infected individuals (Table 1). No predetermined criteria was used to choose patients for phylogenetic sequencing. RNA or DNA was extracted using Qiagen kit QIAAMP DSP VIRUS KIT or AllPrep DNA/RNA, respectively, according to manufacturer’s instructions. Fifteen microliters of RNA and DNA (equal to 100,000 cells) were used as input for the first step PCR using SuperScript III RT/Platinum or and Taq High Fidelity Enzyme Mix (Invitrogen). RNA was reverse transcribed using the reverse primer Rev7659-86 5′ TGGAGAAGTGAATTATATAAATATAAAG [Hxb2 7659←7686]. The 1st round PCR was performed in a 50 µl reaction (0.5 µl (= 5U) High Fidelity Platinum Taq (Life technologies, Darmstadt), 3.5 mM MgCl_2_, 4 µl of dNTPs and (2.0 mM of each) forward For6435-67 5′ ACACATGCCTGTGTACCCACAGACCCCAACCCA) [Hxb2 6435→6467] and reverse Rev7659-86 primers, at 95°C for 10 min followed by 45 cycles (94°C-30 s, 55°C-30 s, 68°C-3 min) and 7 min at 68°C. The 2nd round PCR was performed with 5 µl of first round PCR product in a 50 µl reaction using the same conditions and the primers For6540-62 5′GAGGATATAATCAGTTTATGGGA [Hxb2 6540→6562] and Rev7647-68 5′CACTTCTCCAATTGTCCCTCAT [Hxb2 7647←7668]. To the nested primers were extended with tags that conferred unique identification codes to each plasma or cell fraction. Tagged amplified products were purified using the Ampure beads (Angencourt). Following assessment of DNA concentration (Nanodrop and Qubit) and fragments size (Fragment Analyser, AAT) amplicons were pooled on an equimolar basis. The ends of the DNA were repaired as described by Pacific Bioscience prior to generation of the SMRTbell library (SMRTbell library kit, Pacific Bioscience), which was then purified, quantified and analyzed for fragment size. The library was annealed to sequencing primers at values predetermined by the Binding Calculator (PacBio) and a complex made with the DNA Polymerase (P6/C4 chemistry). The complex was bound to Magbeads and used to set up the required number of SMRT cells for the project. Sequencing was performed using 360-min movie times on the Pacific Biosciences RS11 instrument. Consensus sequences from PacBio reads were generated with accepting only reads where the DNA template had been read at least three times to ensure high quality. All reads were blasted against HIV and converted into fasta files. The resulting sequences were de-multiplexed according to the tags associated with each subcellular fraction and clustered upon 97% identity to exclude differences introduced during PCR amplification. An average of 2,600 reads was obtained ranging between 600 and 5,300 reads. The phylogenetic relationship was inferred by the Maximum Likelihood method based on the General Time Reversible substitution model (GTR + G) and the nucleotide variation within or between plasma and cellular virus pools was estimated using the Gama distributed kimura-two-parameter model and the Mega software package.

### Accession Number(s)

Sequences were submitted to GenBank under accession numbers MG755825-MG756598.

### Assessment of T-Cell Activation Marker, HIV Coreceptor and Restriction Factor Expression by Flow Cytometry

Cryopreserved blood mononuclear cells from ART-treated HIV-1-infected individuals were thawed and stained with Aqua LIVE/DEAD stain kit (4°C; 15 min) and then with anti-CD3, anti-CD4, anti-CD45RA, anti-CXCR3, anti-CXCR5, anti-CCR4, anti-CCR6 PE, anti-HLA DR, anti-Ki67, anti-CD27, anti-CCR7, anti-CCR5 and anti-CXCR4 antibodies (4°C; 25 min). Regarding SAMHD1 staining, blood mononuclear cells were surface stained with Aqua LIVE/DEAD stain kit (4°C; 15 min) and then with anti-CD3, anti-CD4, anti-CD45RA, anti-CXCR3, anti-CXCR5, anti-CCR4, anti-CCR6 PE. Cells were then permeabilized (Fixation/Permeabilization Kit; BD; 45 min; 4°C) and incubated with anti-SAMHD1 (4°C; 25 min) antibody and then with a PE-conjugated donkey-antirabbit antibody (4°C; 25 min). Cells were then washed and acquired on LSRII SORP.

### Flow Cytometry

Data were acquired on a LSR SORP four lasers (405, 488, 532, and 633 nm) and were analyzed as previously described (17, 35).

### Mass Cytometry

Mass cytometry experiments were performed as previously described (17). Briefly, cryopreserved LN mononuclear cells isolated from viremic untreated HIV-infected individuals were thawed and resuspended (10^6^ cells/mL) in complete RPMI medium. Cell viability was assessed using cisplatin (50 µM; 5 min at RT; Sigma-Aldrich) quenched with fetal bovine serum. Cells were then incubated (30 min; 4°C) with metal conjugated antibodies, i.e., anti-CD3-170, anti-CD4-115, anti-CD8-145, anti-CD45RA-169, anti-CXCR5-153, anti-CXCR3-154, anti-CCR6-141, and anti-CCR4-149 (Fluidigm/DVS Science), washed and fixed (10 min; RT) with 2.4% PFA. Total cells were identified by DNA intercalation (1 µM Cell-ID Intercalator, Fluidigm/DVS Science) in 2% PFA at 4°C overnight. Labeled samples were assessed by the CyTOF1 instrument that was upgraded to CyTOF2 (Fluidigm) using a flow rate of 0.045 mL/min. Data were analyzed as previously described (17).

### Statistical Analyses

Statistical significance (*P*-values) was either obtained using two-tailed Chi-square analysis for comparison of positive proportions or using one-way ANOVA (Kruskal–Wallis test) followed by Wilcoxon matched-pairs two-tailed signed rank test as previously described (17, 35). Extreme limiting dilution analysis was applied to obtain statistics for frequencies of cells containing replication competent virus as in previous studies (1, 7, 35). Statistical significance for contribution analysis were either obtained from pairwise comparisons of proportion with FDR correction (multiple tests) or from fisher exact test for pairwise comparisons in case of small sample size. When required, Bonferroni’s correction was applied for multiple comparisons. Finally, Spearman rank test was used for correlations.

## Results

In the present study, 9 viremic HIV-infected individuals and 19 aviremic ART-treated HIV-infected individuals were enrolled. LN biopsies were collected for all nine viremic HIV-infected individuals. Among the aviremic ART-treated individuals, 12 LN biopsies and 14 leukapheresis samples were collected, amongst which 7 individuals were matched for blood and LNs (Table 1). The 19 subjects studied had a documented duration of HIV-1 infection between 1.9 and 28.5 years (mean 10.1 years), a duration of ART between 1.8 and 14 years (mean 4.99 years) and viremia levels <20 HIV-1 RNA copies/mL of plasma for at least 12 months (Table 1).

### Characterization of CD4 T Cell Populations

**Figure 1:**
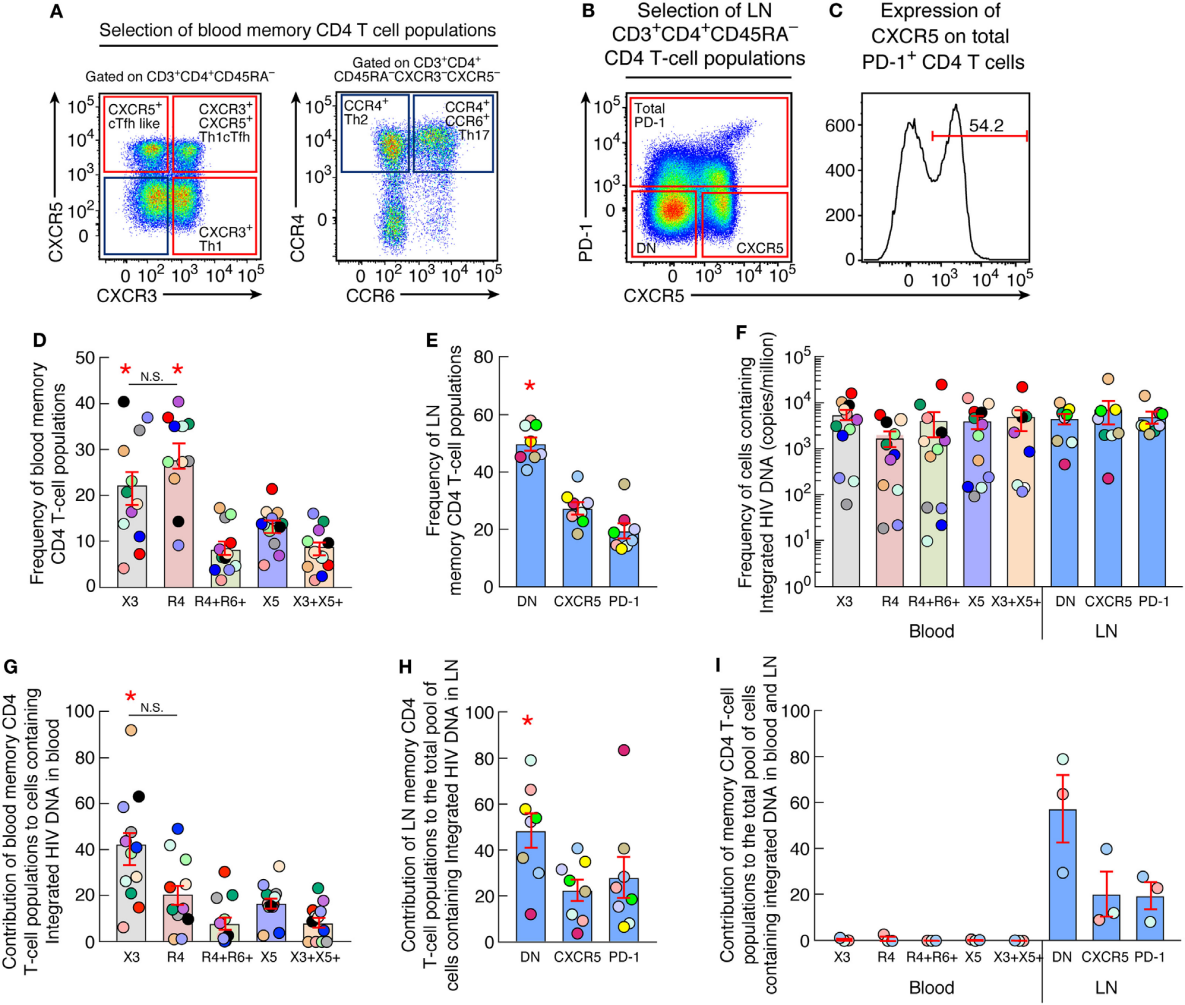
Quantification of HIV Integrated DNA in blood and lymph node memory CD4 T-cell populations **(A)** Representative flow cytometry profile of blood memory (CD45RA^−^) CD4 T-cell populations expressing chemokine receptors isolated from one representative aviremic long-term-treated HIV-1-infected individual. **(B)** Representative flow cytometry profile of lymph nodes (LN) memory (CD45RA^−^) CD4 T-cell populations expressing CXCR5 and/or PD-1 isolated from one aviremic HIV-1-infected long term-treated subject. **(C)** Representative flow cytometry profile of surface expression of CXCR5 on LN PD-1^+^ memory CD4 T cells isolated from one representative aviremic long-term-treated HIV-1-infected individual. **(D)** Frequency of chemokine receptor expressing blood memory (CD45RA^−^) CD4 T cells isolated from aviremic long-term-treated HIV-1-infected individuals (*N* = 12). **(E)** Frequency of CXCR5 and/or PD-1-expressing LN CD4 T cells isolated from aviremic long-term-treated HIV-1-infected individual (*N* = 8). **(F)** Frequency of cells containing integrated HIV DNA (copies per million cells) within chemokine receptor expressing blood (*N* = 12) and CXCR5 and/or PD-1 expressing LN (*N* = 8) memory CD4 T-cell populations. **(G)** Contribution of chemokine receptor expressing blood memory CD4 T-cell populations to the total pool of cells containing integrated HIV DNA in blood (*N* = 12). **(H)** Contribution of CXCR5 and/or PD-1 expressing LN memory CD4 T-cell populations to the total pool of cells containing integrated HIV DNA in LN (*N* = 8). **(I)** Contribution of blood and LN memory CD4 T-cell populations of matched individuals to the total body pool of cells containing integrated HIV DNA in blood and LN compartments (*N* = 3). HIV-infected individuals are color coded **(D–I)**. Histograms correspond to mean of blood or lymph node CD4 T-cell population **(D–I)**; red bars correspond to SEM **(D–I)**. “X3” corresponds to blood CXCR3-expressing CD4 T cells; “R4” corresponds to blood CCR4-expressing CD4 T cells; R4^+^R6^+^ corresponds to blood CCR4^+^CCR6^+^ CD4 T cells; “X5” corresponds to blood CXCR5-expressing CD4 T cells; And X3^+^X5^+^ corresponds to blood CXCR3^+^CXCR5^+^ CD4 T cells. “LN” corresponds to lymph node. Red stars indicate statistical significance (*P* < 0.05). Statistical significance (*P*-values) was obtained using one-way ANOVA (Kruskal–Wallis test) followed by Wilcoxon matched-pairs two-tailed signed rank test **(D–I)**.

The expression of chemokine receptors was first assessed in blood memory CD4 T cells isolated from aviremic ART-treated individuals by multiparametric flow cytometry. To address this issue, blood mononuclear cells were stained with CD3, CD4, CD45RA, CXCR5, CXCR3, CCR4, and CCR6. In parallel, LN mononuclear cells were stained with CD3, CD4, CD45RA, CXCR5, and PD-1 antibodies. Five populations of blood memory (CD45RA^−^) CD4 T cells were identified on the basis of chemokine receptor expression, i.e., CXCR3^+^CXCR5^−^ (CXCR3^+^), CXCR3^−^CXCR5^−^CCR4^+^CCR6^−^ (CCR4^+^), CXCR3^−^CXCR5^−^CCR4^+^CCR6^+^, CXCR3^−^CXCR5^+^ (circulating Tfh-like cells; cTfh) (22, 23), and CXCR3^+^CXCR5^+^ (Th1-like cTfh cells) (28) T cells (Figure 1A; Figure S1 in Supplementary Material). The expression of CCR4 and coexpression of CCR4 and CCR6 was also assessed on the remaining CXCR3^+^, cTfh and Th1-like cTfh cells (Figure S2 in Supplementary Material) and revealed no significant differences between the subpopulations (*P* > 0.05).

Lymph node CD4 T-cell populations were identified on the basis of CXCR5 and PD-1 expression. Because of the very low percentage of PD-1^+^ CD4 T cells in LNs, it was not possible to sort individual PD-1^+^ cell populations and therefore we sorted for the total PD-1^+^ CD4 T cell population, CXCR5^+^PD-1^−^, i.e., single CXCR5^+^ cells and the CXCR5^−^PD^−^1^−^ dual negative cells (DN) cells as previously described (Figure 1B). Of note, PD-1^+^ cells coexpressing CXCR5 represented 61% of the total LN PD-1^+^ CD4 T cell population and were therefore referred to as LN PD-1^+^/Tfh cells (Figure 1C).

### Quantification of Integrated HIV-1 DNA in Sorted Blood and LN CD4 T-Cell Populations

In order to determine the contribution of each CD4 T-cell subset to the pool of HIV-1-infected cells, the percentage of each blood and LN CD4 T cell population on memory (CD45RA^−^) CD4 T cells and the frequency HIV-1-infected cells containing integrated HIV-1 DNA in blood and LN were determined. In blood, CCR4^+^ and CXCR3^+^ CD4 T cell subsets represented the majority, i.e., up to 29 and 22% of the memory CD4 T cells, respectively (Figure 1D) followed by cTfh CD4 T cells that represented 13.7% and finally CCR4^+^CCR6^+^ and Th1-like cTfh CD4 T-cell subsets that represented 8.9 and 8.7% of the memory CD4 T cells, respectively. Regarding the LN compartment, LN DN memory CD4 T cell subset represented up to 50% of the LN memory CD4 T cells followed by the single CXCR5^+^ and PD-1^+^/Tfh cells that represented 27 and 20% of the memory CD4 T cells, respectively (Figure 1E).

The estimation of frequencies of cells containing integrated HIV-1 DNA among the sorted blood (*N* = 12) and LN memory CD4 T-cell populations (*N* = 8) (Figure 1F) revealed no significant differences among memory CD4 T cell populations isolated from blood and/or LN (*P* > 0.05) (Figure 1F). In particular, the frequency of blood CXCR3^+^ CD4 T cells containing integrated HIV-1 DNA reached 5,188 copies per million, 3,882 copies per million in cTfh cells, 4,765 copies per million in Th1-like cTfh cells, 1,732 copies per million CCR4^+^ CD4 T cells, and 3,965 copies per million in CCR4^+^CCR6^+^ subset of CD4 T cells (Figure 1F). Integrated HIV-1 DNA was detected at a mean frequency of 5,281 copies per million cells within LN PD-1^+^/Tfh cells CD4 T cells with no significant differences with the LN DN and single CXCR5 CD4 T-cell populations (*P* > 0.05) (Figure 1F).

We next evaluated the contribution of blood and LN CD4 T-cell populations to the total pool of cells containing integrated DNA within these compartments. The cumulative data indicated that blood CXCR3^+^ CD4 T cells contributed the most and represented about 40% of the blood reservoir containing integrated HIV DNA (Figure 1G). In the LN compartment, DN CD4 T-cell population contributed the most, reaching up to 48% of the LN reservoir containing integrated HIV DNA (*P* < 0.05) (Figure 1H). Finally, we evaluated the overall contribution of each sorted blood and LN CD4 T-cell population to the total pool of cells containing integrated HIV DNA within both blood and LN compartments in matched individuals (*N* = 3). The cumulative data showed that the DN CD4 T-cell population obtained from LNs contributed the most and up to 58% to the total HIV integrated DNA reservoir from blood and LN compartments (Figure 1I).

### HIV Replication and Production of Blood and LN Memory CD4 T Cell Populations

In order to estimate the frequencies of HIV-1-infected cells containing inducible replication competent virus in both blood and LN compartments, we performed a viral outgrowth assay (VOA) on each isolated blood and LN CD4 T cell population (Figure S3 in Supplementary Material). However, the limited number of cells available prevented us to perform the quantitative virus outgrowth assay (Q-VOA) under conventional experimental conditions, i.e., different cell dilutions and multiple replicates, necessary to generate the frequencies of cells containing replication competent and/or infectious virus within the different memory CD4 T-cell populations for each HIV-1-infected individual. Therefore, a conventional VOA was performed using four single replicate cell dilutions, i.e., 5 × 10^5^, 10^5^, 2 × 10^4^, and 4 × 10^3^ cells for all the sorted blood chemokine expressing CD4 T-cell populations (*N* = 13) and three single replicate cell dilutions, i.e., 10^5^, 2 × 10^4^, and 4 × 10^3^ cells for all the sorted LN CD4 T-cell populations sorted on the basis of CXCR5 and PD-1 expression (*N* = 11). All cell populations were stimulated with anti-CD3 and anti-CD28 monoclonal antibodies (MAbs) and cultured with allogeneic CD8-depleted blood mononuclear cells isolated from HIV-uninfected individuals for 14 days (Figure S3 in Supplementary Material). The presence of HIV-1 RNA was assessed in the culture supernatants at day 14 as previously described (17). Both the proportion of positive wells and the levels of HIV-1 RNA were generated in the VOA using the highest common concentration of cells (10^5^ cells/condition), while the limiting dilution format was used to evaluate the frequencies of cells containing inducible replication competent virus as in previous studies (35). The frequencies of cells containing inducible replication competent virus assessed by the detection of HIV RNA at day 14 in VOA supernatants was expressed as RNA-unit per million (RUPM) (32) (Figure S3 in Supplementary Material).

**Figure 2:**
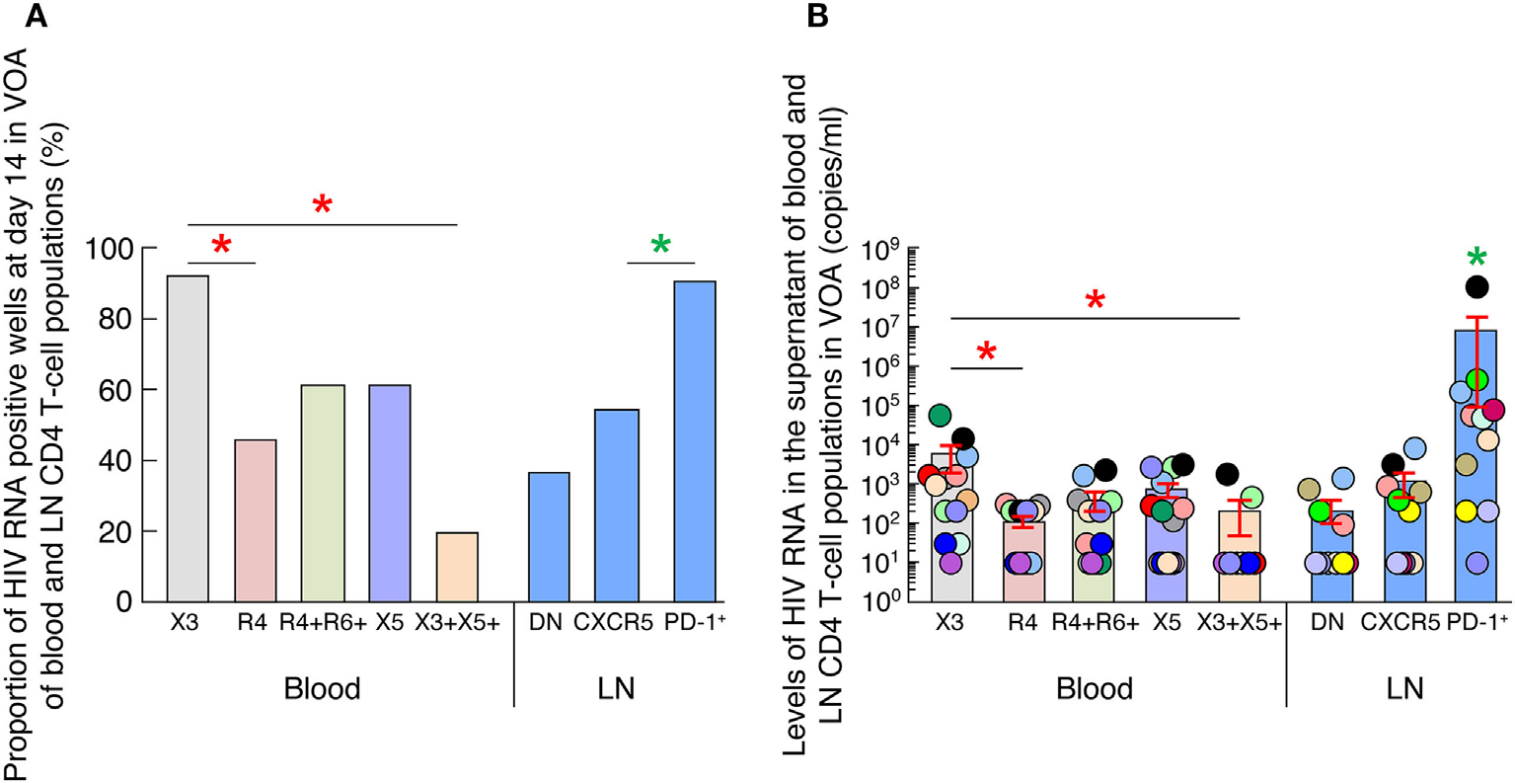
HIV replication and production of blood and lymph node (LN) memory CD4 T cell populations. **(A)** Proportion of HIV RNA positive wells among blood (*N* = 13) and LN (*N* = 11) memory CD4 T-cell populations at day 14 of virus outgrowth assay (VOA). Wells with detectable HIV-1 RNA (≥20 HIV-1 RNA copies/mL) were referred to as HIV-1 RNA-positive wells. **(B)** Levels of HIV-1 RNA in blood (*N* = 13) and LN (*N* = 11) memory CD4 T cell populations at day 14 of VOA. Undetectable values were arbitrarily defined as 10 HIV-1 RNA copies/mL. HIV-infected individuals are color coded (C). “X3” corresponds to blood CXCR3-expressing CD4 T cells; “R4” corresponds to blood CCR4-expressing CD4 T cells; R4^+^R6^+^ corresponds to blood CCR4^+^CCR6^+^ CD4 T cells; “X5” corresponds to blood CXCR5-expressing CD4 T cells; And X3^+^X5^+^ corresponds to blood CXCR3^+^CXCR5^+^ CD4 T cells. “LN” corresponds to lymph node. Red stars indicate statistical significance with in blood compartment (*P* < 0.05). Green stars indicate statistical significance within LN compartment (*P* < 0.05). Statistical significance (*P*-values) was either obtained using two-tailed Chi-square analysis for comparison of positive proportions **(A)** or using one-way ANOVA (Kruskal–Wallis test) followed by Wilcoxon matched-pairs two-tailed signed rank test **(B)**.

We first compared the proportion of HIV RNA positive wells at day 14 in the VOA culture supernatants of all blood (*N* = 13) and LN CD4 T-cell populations (*N* = 11) at the highest common cell concentration (i.e., 10^5^) (Figure 2A) (35). Of note, 6 individuals were matched for blood and LN. The data showed that wells containing LN PD-1^+^/Tfh CD4 T cells were significantly more frequently scored positive (90% of positive wells, corresponding to 9 out of the 11 individuals tested) as compared to LN CXCR5^+^ and LN DN CD4 T cell populations (*P* < 0.05) (Figure 2A), supporting previous observation (17). However, in blood, wells containing CXCR3-expressing CD4 T cells were more frequently scored positive (92% of positive wells, corresponding to 12 out of 13 individuals tested) than any other blood CD4 T-cell populations (Figure 2A).

We next assessed the levels of HIV-1 RNA in culture supernatants of all blood (*N* = 13) and LN (*N* = 11) CD4 T-cell populations at the highest common cell concentration (i.e., 10^5^) cells at day 0 and 14 (Figure 2B) (35). Of note, none of the culture supernatants collected at day 0 were positive for the detection of HIV-1 RNA (data not shown). Consistent with previous study (17), LN PD-1^+^/Tfh cell VOA culture supernatants contained significantly higher levels of HIV-1 RNA than any other LN memory CD4 T-cell populations (*P* < 0.05) (Figure 2B).

However, in blood, CXCR3-expressing CD4 T cells VOA culture supernatants contained higher levels of HIV-1 RNA than any other blood memory CD4 T-cell populations (except from cTfh cells; *P* = 0.08) (Figure 2B), suggesting that blood CXCR3-expressing CD4 T cells of ART-treated aviremic HIV-1 infected individuals might be enriched in cells containing replication competent virus.

### Blood CXCR3-Expressing CD4 T Cells of Long-term-Treated Aviremic HIV-1-Infected Individuals Are Enriched in Cells Containing Replication Competent Virus

**Figure 3:**
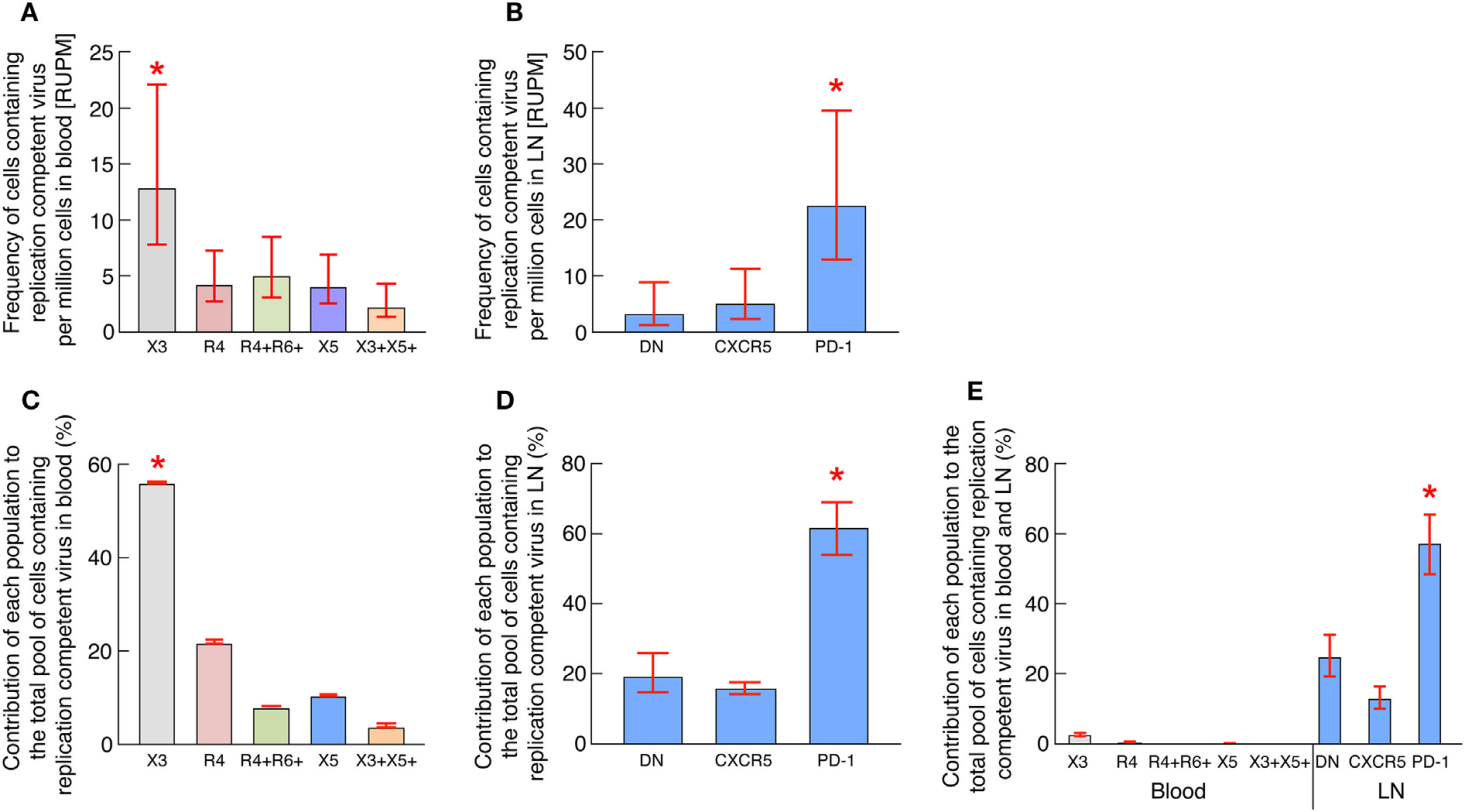
Blood CXCR3-expressing CD4 T cells of long-term-treated aviremic HIV-1-infected individuals are enriched in cells containing replication competent virus. **(A)** Estimated RNA-unit per million (RUPM) frequencies in chemokine receptor expressing blood memory CD4 T-cell populations (*N* = 13). **(B)** Estimated RUPM frequencies in CXCR5 and/or PD-1 expressing lymph node (LN) memory CD4 T-cell populations (*N* = 11). **(C)** Estimated contribution of blood memory CD4 T-cell populations to the pool of cells containing replication competent virus in blood (*N* = 13). **(D)** Estimated contribution of LN memory CD4 T-cell populations to the pool of cells containing replication competent virus in LN compartment (*N* = 11). **(E)** Estimated contribution of blood and LN memory CD4 T-cell populations of matched individuals to the total pool of cells containing replication competent virus in blood and LN compartments (*N* = 6). Contribution of memory CD4 T-cell populations to the pool of cells containing replication competent virus was calculated as previously described (17). Histograms correspond to estimated mean **(A–E)** and red bars correspond to the lower and upper confidence interval at 0.95 **(A–E)**. “X3” corresponds to blood CXCR3-expressing CD4 T cells; “R4” corresponds to blood CCR4-expressing CD4 T cells; R4^+^R6^+^ corresponds to blood CCR4^+^CCR6^+^ CD4 T cells; “X5” corresponds to blood CXCR5-expressing CD4 T cells; And X3^+^X5^+^ corresponds to blood CXCR3^+^CXCR5^+^ CD4 T cells. “LN” corresponds to lymph node. Red stars indicate statistical significance (*P* < 0.05). Statistical significance (*P*-values) was either obtained using Extreme Limiting Dilution analysis (http://bioinf.wehi.edu.au/software/elda/) **(A,B)**, by pairwise comparisons of proportion with FDR correction (multiple tests) **(C,E)** or by Fisher’s exact test for pairwise comparisons **(D)** (36).

To address the issue of the average frequency of HIV-1-infected cells containing inducible replication competent virus for each memory CD4 T-cell population in blood and LN compartments, the data generated from the single replicate cell dilutions of blood and LN CD4 T-cell populations using the conventional VOA were pooled together such that each patient well at a particular tested concentration in the VOA now represented a replicate; mean frequencies within the cohort were therefore then estimated using extreme limiting dilution assay (33) as previously described (17). The frequencies of HIV-1-infected cells containing inducible replication competent virus are expressed in average RNA-unit per million (RUPM) (32) (Figures 3A,B) and statistical significance was obtained following a pair-wise test performed using Extreme Limiting Dilution Assay (33). Consistent with previous study (17), LN PD-1^+^/Tfh cells were significantly enriched in cells containing replication competent virus reaching 23 cells per million (Figure 3B). Interestingly however, the results obtained from blood samples indicated that blood CXCR3-expressing CD4 T cells but not CXCR5-expressing CD4 T cells were significantly enriched in cells containing inducible replication competent virus reaching about 13 cells per million (*P* < 0.05) (Figure 3A) while CXCR5-expressing CD4 T cells reached around 4 cells per million.

On the basis of the above data and since various CD4 T-cell populations may contribute to the total pool of cells containing replication competent virus within blood and LN compartments, the assessment of the relative contribution of each CD4 T cell population was then performed using (1) the frequency of each cell population in each compartment (blood and/or LN), (2) the estimated numbers of CD4 T lymphocytes in different lymphoid organs (34), and (3) the estimated frequencies of cells containing inducible replication competent virus within each blood and LN memory CD4 T cell population as previously described (17). The results indicated that in blood, the CXCR3-expressing CD4 T cells contributed the most to the pool of cells containing inducible replication competent virus in blood and represented about 56% of the blood reservoir containing replication competent virus (*P* < 0.05) (Figure 3C). Consistent with previous study (17), LN PD-1^+^/Tfh cells contributed the most to the pool of cells containing inducible replication competent virus in LN (represented about 60%) (*P* < 0.05) (Figure 3D) and contributed the most to the pool of cells containing inducible replication competent virus in blood and LN (represented about 58%) (*P* < 0.05) (Figure 3E).

Taken together, these data indicate that blood CXCR3-expressing CD4 T cells isolated from ART-treated aviremic HIV-1-infected individuals are enriched in cells containing inducible replication competent virus and contributed the most to the HIV reservoir in blood.

### Blood CXCR3-Expressing CD4 T Cells of ART-Treated Aviremic HIV-1-Infected Individuals Represent the Major Source of Infectious HIV-1 in Blood

**Figure 4:**
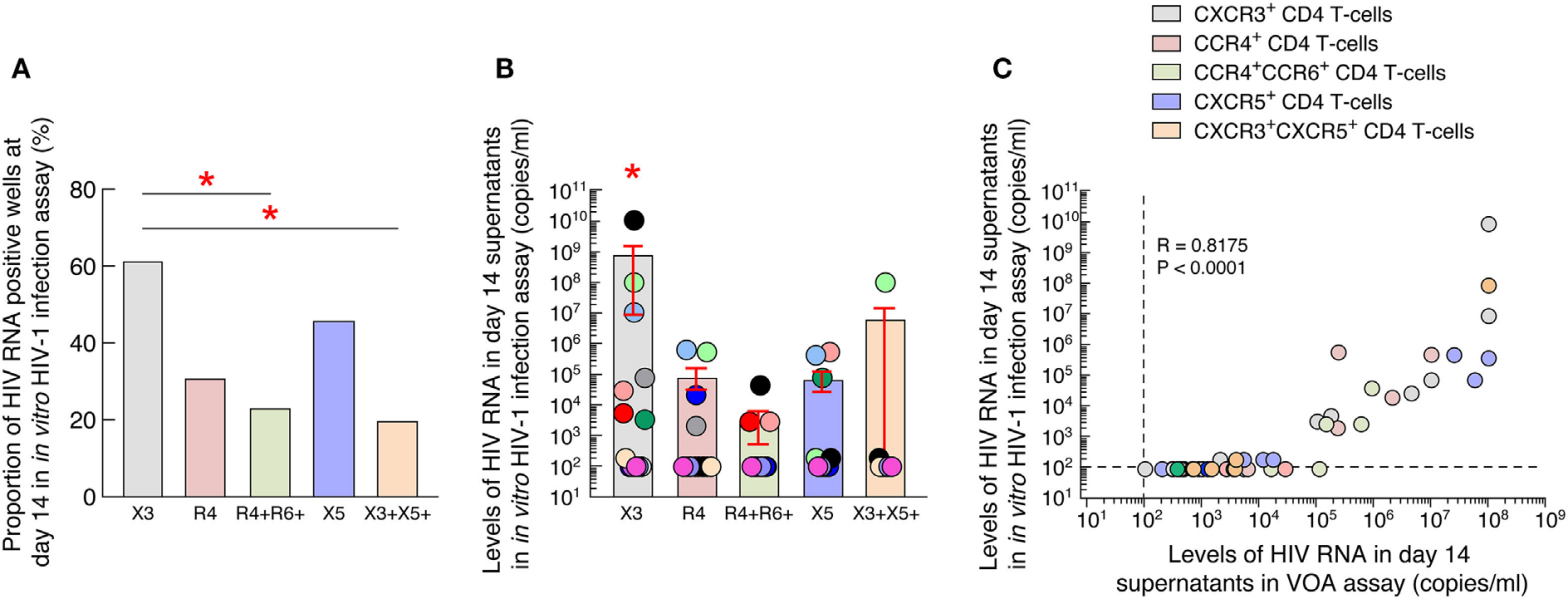
Blood CXCR3^+^ CD4 T cells of antiretroviral therapy (ART)-treated aviremic HIV-1-infected individuals represent the major source of infectious HIV-1 in blood. **(A)** Proportion of HIV-1-infected individuals with detectable HIV-1 RNA (≥200 HIV-1 RNA copies/mL) in the *in vitro* HIV-1 infection assay. **(B)** Levels of HIV-1 RNA in blood memory CD4 T cell populations at day 14 of *in vitro* HIV-1 infection assay (*N* = 13). **(C)** Correlation between HIV-1 RNA levels measured in VOA and in *in vitro* HIV-1 infection assay in supernatants of blood memory CD4 T-cell populations (*N* = 13). Undetectable values were arbitrarily defined as 100 HIV-1 RNA copies/mL **(B,C)**. Each individual is uniquely color coded in **(B)**. Each CD4 T-cell population is uniquely colored in **(C)**. Histograms correspond to mean **(A,B)**; red bars correspond to SEM **(B)**. “X3” corresponds to blood CXCR3-expressing CD4 T cells; “R4” corresponds to blood CCR4-expressing CD4 T cells; R4^+^R6^+^ corresponds to blood CCR4^+^CCR6^+^ CD4 T cells; “X5” corresponds to blood CXCR5-expressing CD4 T cells; And X3^+^X5^+^ corresponds to blood CXCR3^+^CXCR5^+^ CD4 T cells. Red stars indicate statistical significance (*P* < 0.05) **(A,B)**. Statistical significance (*P*-values) was either obtained using two-tailed Chi-square analysis for comparison of positive proportions **(A)** or using one-way ANOVA (Kruskal–Wallis test) followed by Wilcoxon matched-pairs two-tailed signed rank test **(B)** or Spearman rank test for correlation **(C)**.

In order to determine whether the virus obtained in the VOA culture supernatants of blood memory CD4 T-cell populations was infectious, we performed an *in vitro* HIV-1 infection assay. For these purposes, day 14 VOA culture supernatants of the highest cell concentration (i.e., 5 × 10^5^) were used to inoculate preactivated CD8-depleted blood mononuclear cells isolated from HIV negative individuals as previously described (17). Culture supernatants were collected at day 0 and 14 and assessed for the presence of HIV-1 RNA. Of note, none of the culture supernatants collected at day 0 had detectable levels of HIV-1 RNA (data not shown). However, after 14 days of culture, HIV-1 RNA was more frequently detected in culture supernatants of CXCR3-expressing CD4 T cells (8 out of the 13 ART-treated aviremic HIV-1-infected individuals tested) as compared to any other blood CD4 T-cell populations (Figure 4A). In addition, the levels of HIV-1 RNA detected in culture supernatants of blood CXCR3-expressing CD4 T cells were significantly higher as compared to any other blood CD4 T-cell subsets (*P* < 0.05) (Figure 4B). Finally, we determined the relationship between HIV-1 RNA levels detected in the VOA and those detected in the *in vitro* HIV-1 infection assay. The results indicated a strong correlation between HIV-1 RNA levels detected in VOA culture supernatants and HIV-1 RNA levels detected in the *in vitro* HIV-1 infection assay (*r* = 0.8175, *P* < 0.0001) (Figure 4C).

Taken together, these data suggest that blood CXCR3-expressing CD4 T cells of ART-treated aviremic HIV-1-infected individuals represent the major source of infectious HIV-1 in blood.

### Phylogenetic Sequence Analyses of the Highly Variable EnvV1V4 Region in Sorted Blood and LN Memory CD4 T-Cell Populations

In order to address the phylogenetic relationship between HIV sequences obtained from blood and LN CD4 T-cell populations, we sorted blood and LN CD4 T-cell populations of matched ART-treated HIV-infected individuals as previously mentioned and single genome sequencing of the highly variable region of the gp160 virus envelope, the EnvV1V4 region, was performed using next generation sequencing platform from PacBio Systems as previously described (37).

**Figure 5:**
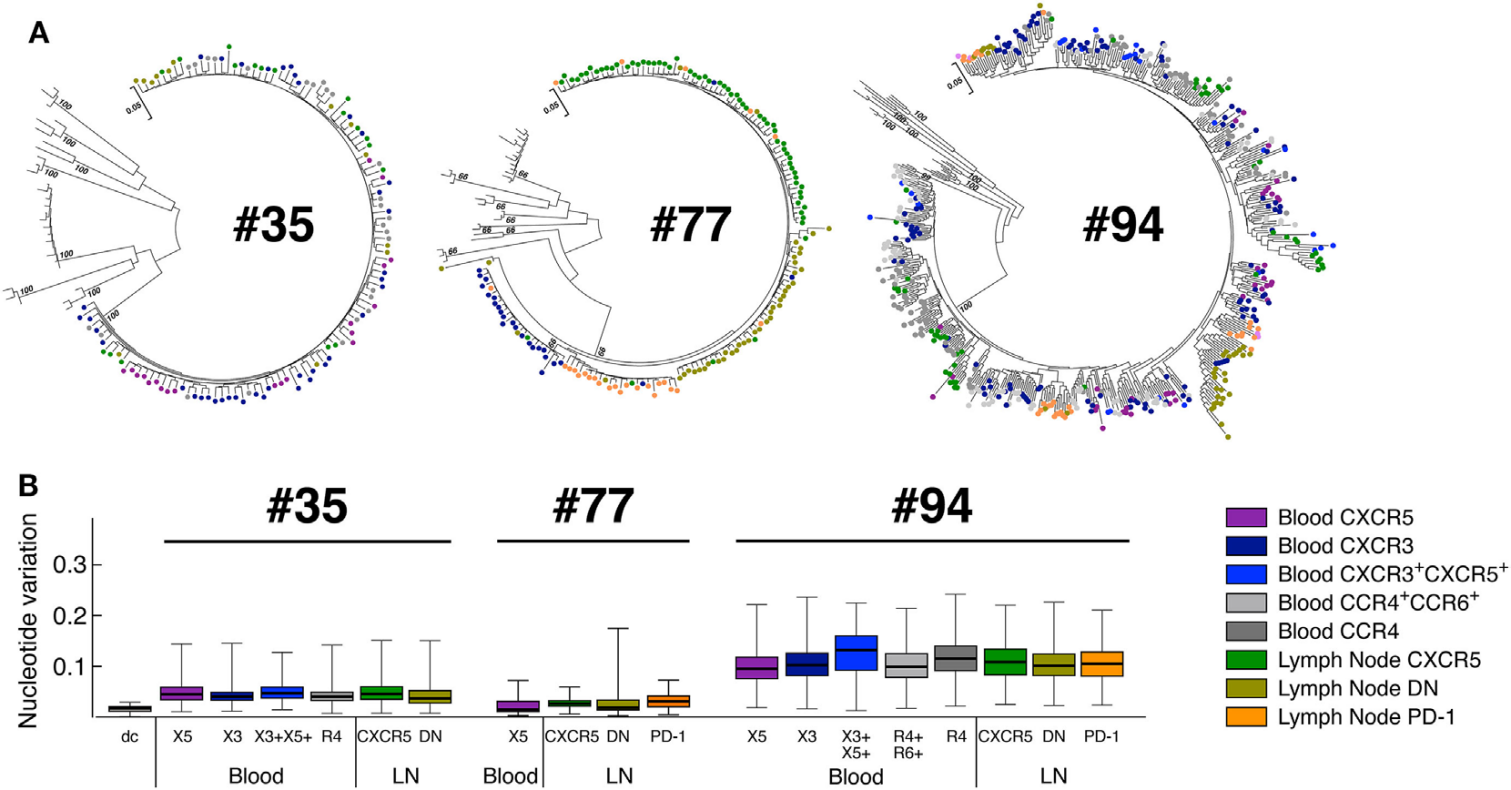
Phylogenetic relationship of HIV-1 envelope sequences derived from CD4 T-cell populations. Sequences of the highly variable EnvV1V4 region (Hxb 6540-7668) were derived from blood and LN memory CD4 T-cell populations isolated from aviremic-treated HIV-1-infected individuals (*N* = 3). Virus quasi-species were amplified and sequenced [single molecule, real-time (SMRT) method/PacBio Systems]. **(A)** Phylogenetic relationship of HIV-1 envelope sequences derived from CD4 T-cell populations (*N* = 3). The phylogenetic relationship was inferred by the Maximum Likelihood method based on the General Time Reversible substitution model (GTR + G). Each tree includes reference sequences from subtype B and non-B HIV-1-infected individuals. **(B)** Nucleotide variations observed within each blood and LN memory CD4 T-cell population estimated using the Gama distributed kimura-two-parameter. CD4 T-cell populations were color coded **(A,B)**. dc corresponds to the laboratory isolate control used to monitor sequence diversity induced by the method. “X3” corresponds to blood CXCR3-expressing CD4 T cells; “R4” corresponds to blood CCR4-expressing CD4 T cells; R4^+^R6^+^ corresponds to blood CCR4^+^CCR6^+^ CD4 T cells; “X5” corresponds to blood CXCR5-expressing CD4 T cells; And X3^+^X5^+^ corresponds to blood CXCR3^+^CXCR5^+^ CD4 T cells. “LN” corresponds to lymph node.

The phylogenetic analysis revealed that the sequences obtained from the individual #35 and #077 were monophyletic while those obtained from individual #094 were not (Figure 5A), indicating varying degree of virus diversity. In all three ART-treated HIV-infected individuals tested, phylogenetic inference revealed that virus pools from any given cell population were closely related to the virus from all other cell fractions demonstrating a lack of virus compartmentalization (Figure 5B).

### PD-1 Expression on Blood CXCR3-Expressing CD4 T Cells Positively Correlates with the Levels of HIV-1 RNA Produced in the VOA Culture Supernatants

**Figure 6:**
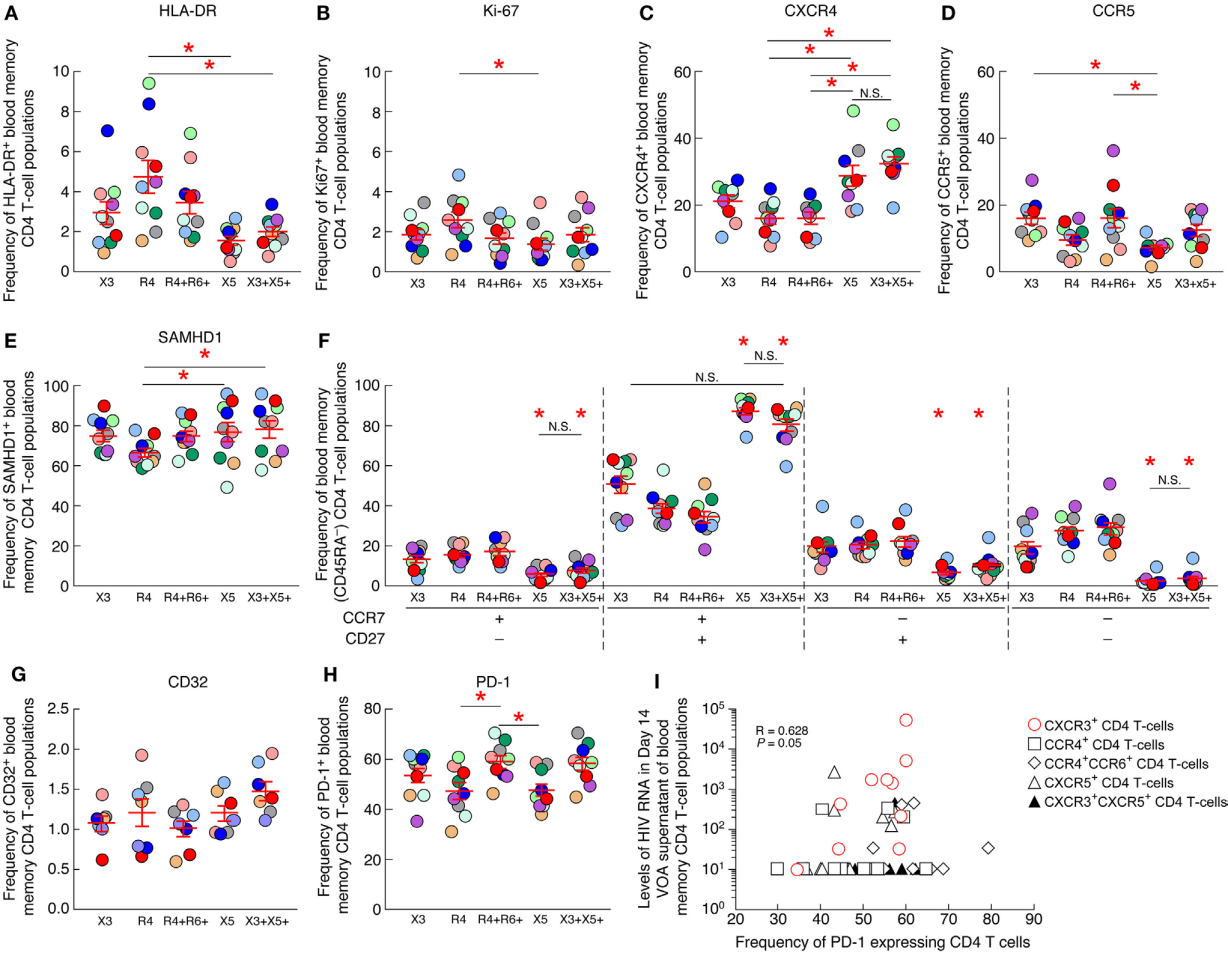
PD-1 expression on blood CXCR3^+^ CD4 T cells positively correlates with the levels of HIV-1 RNA produced in the virus outgrowth assay (VOA) culture supernatants. Percentage of expression of HLA-DR **(A)**, Ki-67 **(B)**, CXCR4 **(C)**, CCR5 **(D)**, SAMHD1 **(E)** on chemokine receptor expressing blood memory (CD45RA^−^) CD4 T-cell populations isolated from aviremic long-term-treated HIV-1-infected individuals (*N* = 10). **(F)** Differentiation profile of chemokine receptor expressing blood memory (CD45RA^−^) CD4 T-cell populations isolated from aviremic long-term-treated HIV-1-infected individuals (*N* = 10). Percentage of CD32^+^ (*N* = 7) **(G)** or PD-1^+^ (*N* = 10) **(H)** chemokine receptor expressing blood memory (CD45RA^−^) CD4 T-cell populations isolated from aviremic long-term-treated HIV-1-infected individuals. **(I)** Correlation between HIV-1 RNA levels detected in day 14 VOA supernatants of chemokine receptor expressing blood memory CD4 T-cell populations and the percentage of PD-1-expressing CD4 T cells within each chemokine receptor expressing blood memory CD4 T-cell population. Undetectable values were arbitrarily defined as 10 HIV-1 RNA copies/mL **(I)**. Red bars correspond to SEM **(A–H)**. “X3” corresponds to blood CXCR3-expressing CD4 T cells; “R4” corresponds to blood CCR4-expressing CD4 T cells; R4^+^R6^+^ corresponds to blood CCR4^+^CCR6^+^ CD4 T cells; “X5” corresponds to blood CXCR5-expressing CD4 T cells; And X3^+^X5^+^ corresponds to blood CXCR3^+^CXCR5^+^ CD4 T cells. Each HIV-infected individual was color coded **(A–H)**. Red stars indicate statistical significance (*P* < 0.05) **(A–H)**. Statistical significance (*P*-values) was either obtained using one-way ANOVA (Kruskal–Wallis test) followed by Wilcoxon matched-pairs two-tailed signed rank test **(A–H)** or Spearman rank test for correlation **(I)**.

Next, we explored whether specific parameters such as the level of T-cell activation (assessed by HLA-DR and Ki-67 expression), T-cell differentiation (assessed by CD45RA, CCR7 and/or CD27 expression), HIV coreceptor (CCR5 and CXCR4) or restriction factor (SAMHD1) expression may be associated with the increased frequency of cells containing replication competent virus within blood CXCR3-expressing CD4 T cells. To address this issue, the level of HLA-DR, Ki-67, CCR5, CXCR4, SAMHD1, CD45RA, CCR7, and CD27 were determined on the various blood CD4 T-cell populations of HIV-1-infected ART-treated individuals by flow cytometry (Figures 6A–F). The cumulative data indicated that blood CXCR3-expressing CD4 T cells were not significantly enriched in activated T cells expressing HLA-DR or Ki-67 (*P* > 0.05) (Figures 6A,B), in cells expressing the HIV coreceptors CXCR4 or CCR5 (*P* > 0.05) (Figures 6C,D). In addition, blood CXCR3-expressing CD4 T cells were not significantly enriched in central memory (CCR7^+^CD27^+^) nor in transitional memory (CCR7^−^CD27^+^) CD4 T cells (*P* > 0.05) (Figure 6F). Finally, blood CXCR3-expressing CD4 T cells did not express significantly lower levels of the host restriction factor SAMHD1 (*P* > 0.05) (Figure 6E).

Since, a recent study highlighted that blood CD32-expressing CD4 T cells were enriched in HIV-infected cells (12), therefore we assessed the level of CD32 expression on chemokine expressing CD4 T-cell populations (Figure 6G). The cumulative data indicated that blood CXCR3-expressing CD4 T cells were not significantly enriched in cells expressing CD32 (*P* > 0.05) (Figure 6G).

Finally, since PD-1 expression on CD4 T cells has been associated with increased frequencies of HIV-infected cells (16, 17), we next assessed the level of PD-1 expression on each blood memory CD4 T-cell population of ART-treated HIV-infected individuals. The cumulative data indicated that blood CXCR3-expressing CD4 T cells were not significantly enriched in cells expressing PD-1 (*P* > 0.05) (Figure 6H), however, the frequency of PD-1 expressing blood CXCR3-expressing CD4 T cells positively correlated with the levels of HIV-1 RNA produced in the VOA culture supernatants of CXCR3-expressing CD4 T cells (*r* = 0.628, *P* = 0.05) (Figure 6I), suggesting that PD-1^+^CXCR3^+^ CD4 T cells might be enriched in cells containing inducible replication competent virus in blood.

### LN PD-1 Expressing CD4 T Cells Express High Levels of CXCR3

**Figure 7:**
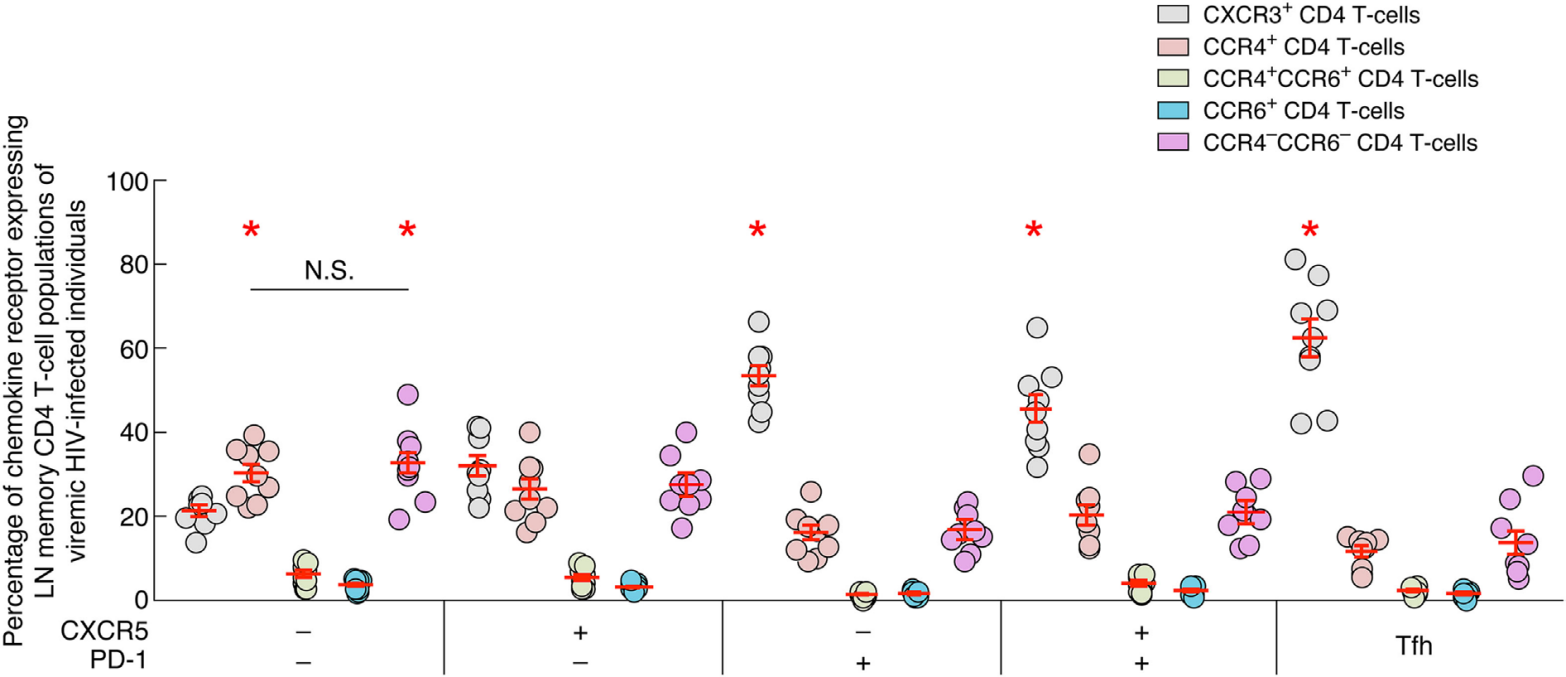
Chemokine receptor expression on lymph node memory CD4 T cell populations of viremic HIV-infected individuals. Percentage of chemokine receptor expression on lymph nodes (LNs) CD4 T-cell populations of viremic HIV-infected individuals (*N* = 9). Red bars correspond mean ± SEM. CD4 T-cell populations were color coded. Red stars indicate statistical significance (*P* < 0.05). Statistical significance (*P*-values) was either obtained using one-way ANOVA (Kruskal–Wallis test) followed by Wilcoxon matched-pairs two-tailed signed rank test.

In order to determine the potential origin of blood CXCR3-expressing CD4 T cells, the expression of CXCR3, CCR4 and/or CCR6 was assessed on LN memory (CD45RA^−^) CD4 T-cell populations isolated from viremic untreated HIV-infected individuals (Figure 7). The data showed that all PD-1-expressing LN CD4 T-cell populations of viremic HIV-infected individuals were enriched in CXCR3^+^ CD4 T cells in comparison to CCR4^+^, single CCR6^+^, CCR4^+^CCR6^+^, and CCR4^−^CCR6^−^ CD4 T cells (*P* < 0.05) (Figure 7). In contrast the PD-1-negative CD4 T-cell populations expressed either comparable levels of CXCR3 and CCR4 (single CXCR5 LN CD4 T cells; *P* > 0.05) or were dominated by CCR4-expressing and CCR4^−^CCR6^−^ CD4 T cells (DN LN CD4 T cells; *P* < 0.05) (Figure 7).

Taken together, these data suggest that CXCR3-expressing blood CD4 T cells may originate from LN PD-1^+^ CD4 T cells. However, further longitudinal analyses would be needed to support this hypothesis.

## Discussion

One of the major barriers to HIV eradication is the presence of cells containing latent, transcriptionally silent, but inducible replication competent virus in ART-treated aviremic HIV-infected individuals (4, 8, 38). Therefore, great effort was placed to characterize HIV-infected cells in blood and tissues (39). In this regard, we have recently shown that LN PD-1^+^/Tfh cells were enriched in cells containing inducible replication competent virus in ART-treated aviremic HIV-infected individuals (17), which is probably associated with the reduced capacity of CD8 T cells and cART to penetrate into germinal center (GC) areas (19, 40).

Since memory LN Tfh cells may egress from GCs and recirculate in blood (20), we hypothesized that the LN Tfh cell counterpart recirculating in blood might also be enriched in HIV-infected cells in ART-treated aviremic HIV-1-infected individuals. We therefore determined the frequency of cells containing integrated HIV-1 DNA or replication competent virus and their contribution to the HIV reservoir in blood and LN CD4 T-cell populations defined by chemokine receptor expression. Since the blood circulating Tfh counterpart has usually been identified on the basis of expression of chemokine receptors CXCR5 (22) and/or CXCR3 (20), we have first gated on and sorted for CXCR5 and/or CXCR3-expressing memory CD4 T-cell populations. In addition, cells expressing neither CXCR5 nor CXCR3, have previously been shown to be enriched in TH2-like (expressing CCR4^+^) and TH17-like (expressing CCR4 and CCR6) cells (41). Therefore, in addition to the CXCR5^+^ and/or CXCR3^+^ cells, we chose to sort for CCR4^+^ and CCR4^+^CCR6^+^ subsets amongst the CXCR3 and CXCR5 dual negative cells. Of note, the three other remaining populations, i.e., CXCR3^+^, CXCR5^+^, and CXCR3^+^CXCR5^+^ cell populations also contained cells expressing CCR4 and/or CCR6, but showed no significant differences between them. Therefore, in the present study, we focused on and compared chemokine receptor expressing blood CD4 T-cell populations including CXCR5^+^ and CXCR5^+^CXCR3^+^ blood memory CD4 T cells, corresponding to “cTfh” (22, 23) and “Th1-like cTfh” (28) CD4 T cells, respectively, CXCR5^−^CXCR3^−^CCR4^+^, CXCR5^−^CXCR3^−^CCR4^+^CCR6^+^ blood memory CD4 T-cell populations and LN CD4 T-cell populations including LN PD-1^+^/Tfh CD4 T cells.

Interestingly, we did not observe significant differences in terms of frequencies of cells containing HIV-1 integrated DNA within and across blood and LN compartments. However, we showed that the blood CXCR3-expressing CD4 T cells contributed the most (up to 56%) to the HIV reservoir in blood, while DN LN CD4 T cells contributed the most to the HIV reservoir in LN.

We then evaluated and compared the capacity of blood and LN memory CD4 T-cell populations isolated from ART-treated aviremic HIV-1-infected individuals to support active virus replication and produce infectious viruses. In this context, we showed that, consistent with previous studies, LN PD-1^+^/Tfh cells were the largest source of inducible replication competent virus. Interestingly, we showed that, in blood, CXCR3-expressing CD4 T cells but not CXCR5-expressing CD4 T cells were enriched in inducible replication competent and infectious virus and contributed the most to the replication competent reservoir in blood.

Unfortunately, the results obtained using the VOA assay did not allow to determine whether CXCR3-expressing CD4 T cells contained more cells with intact provirus or whether the intact provirus was more easily inducible in CXCR3-expressing CD4 T cells as compared to other cell populations. One way to address this issue would be to perform full length sequencing of HIV provirus and to determine HIV provirus integration sites within each CD4 T-cell populations isolated from blood and LN compartments. Interestingly, the recent study by Lee et al. highlighted the presence of higher proportion of intact proviruses within blood type 1 helper (Th1) CD4 T cells (42). Since, blood CXCR3-expressing CD4 T cells are usually enriched in cells harboring Th1 functions (41, 43, 44), it is well possible that blood CXCR3-expressing CD4 T cells would also be enriched in cells containing a higher proportion of intact provirus. Full length sequencing of HIV provirus and HIV integration site determination would also help to determine whether the accumulation of replication competent virus within one particular subset is associated with homeostatic T-cell proliferation or with a higher infection rate of blood CXCR3-expressing CD4 T-cell precursor. Indeed, recent studies have highlighted the role of homeostatic clonal expansion of blood HIV-infected memory CD4 T cells in HIV persistence (42, 45). Notably, Lee et al. also suggested that Th1 cells containing intact provirus may have accumulated in blood through clonal expansion (42).

Furthermore, various other parameters including epigenetic modifications such as DNA methylation of HIV gene promoters or HIV provirus integration site may also potentially contribute to explain the relative lack of inducibility of cells containing intact proviruses (46, 47). In this regard, recent integration site analyses performed on blood CD4 T cells of treated HIV-infected patients demonstrated that intact HIV provirus may be enriched in transcriptionally silent parts of the genome, supporting the relative difficulty to reactivate HIV replication by VOA (46). Therefore, additional studies would be needed to determine the potential mechanism by which HIV-infected cells accumulate within blood CXCR3-expressing CD4 T cells.

To determine the potential origin of blood CXCR3-expressing memory CD4 T cells containing replication competent virus, we performed proviral sequencing of EnvV1V4 region amplified from CD4 T-cell populations isolated from blood and LN compartments. The results obtained indicated that proviral sequences amplified from blood and LN CD4 T-cell populations were intermingled with each other without any indication of compartmentalization. Therefore, these results although limited to certain cell populations indicated that the virus was highly related between the different cell populations, suggesting dynamic interchanges between the two compartments.

We then conducted a series of experiments to determine the potential parameters associated with the enrichment of cells containing replication competent virus within blood CXCR3-expressing CD4 T cells. Notably, blood CXCR3-expressing CD4 T cells did not express significantly higher levels of HIV coreceptors CCR5 and/or CXCR4, were not significantly more activated or did not express significantly lower levels of SAMHD1 restriction factor as compared to the other blood chemokine receptor expressing CD4 T cells. In addition, blood CXCR3-expressing CD4 T cells did not express significantly higher levels of CD32 or PD-1, however, the level of PD-1 expression on blood CXCR3-expressing CD4 T cells directly correlated with the level of HIV RNA produced in the VOA supernatants suggesting that blood CXCR3-expressing CD4 T cells containing replication competent virus might express PD-1.

We then hypothesized that blood CXCR3-expressing CD4 T cells containing replication competent virus might have originated from HIV-infected LN CD4 T cells, that would have been infected during the viremic phase and would recirculate in blood after treatment initiation. Indeed the low state of activation and cell cycle progression of CXCR3-expressing memory CD4 T cells as assessed by the levels of HLA-DR and Ki-67 expression, respectively, also suggests that they may represent those cells that may have exited the tissue sites where prior virus replication may have taken place. Therefore, in order to address the origin of these cells, we assessed the chemokine receptor expression on various LN memory CD4 T cells defined by CXCR5 and PD-1 expression isolated from untreated viremic HIV-infected individuals. Even though the analysis was cross-sectional and not longitudinal, the results obtained indicated that CXCR3 is the most dominant chemokine receptor expressed on PD-1^+^ CD4 T cells including Tfh cells in viremic HIV-infected individuals whereas PD-1-negative CD4 T-cell populations, i.e., DN and single CXCR5 CD4 T-cell populations expressed either comparable levels of CXCR3 and CCR4 or expressed higher levels of CCR4. It is therefore possible that HIV-infected CXCR3-expressing CD4 T cells may originate from HIV-infected PD-1^+^CXCR3^+^ LN CD4 T cells in general and from Tfh cells in particular. Indeed, it has been clearly established that LN GC Tfh cells downregulate CXCR5 expression in order to egress from the B cell follicle post-GC response (48, 49). However, longitudinal assessment of chemokine receptor expression on LN Tfh cells from the viremic phase of infection till the control of infection post-ART treatment may provide further evidence for the chemokine receptors that are retained on these cells to orchestrate further migration from tissues and recirculation in blood.

Taken together, our data highlight the heterogeneity in the CD4 T-cell populations harboring cells containing inducible replication competent virus in blood and LNs of aviremic ART-treated HIV-infected individuals. In particular, we show that blood CXCR3-expressing CD4 T cells but not CXCR5-expressing CD4 T cells were enriched in inducible replication competent virus and contributed the most to the replication competent reservoir in blood. However, additional studies would be needed to determine their potential origins and the mechanism by which HIV-infected cells accumulated within this particular subset.

## Ethics Statement

The present study was approved by the Institutional Review Board of the Centre Hospitalier Universitaire Vaudois, and all subjects gave written informed consent.

## Author Contributions

RB, FP, AR, AN, GP, WP, and MP designed the experiments. RB, FP, AR, and AN performed the experiments. RB, FP, AR, AN, WP, GP, and MP interpreted the data. MC and J-MC provided the samples. RB, GP, and MP wrote the manuscript. All the authors read and approved the final manuscript.

## Conflict of Interest Statement

The authors declare that the research was conducted in the absence of any commercial or financial relationships that could be construed as a potential conflict of interest.

## Abbreviations

ART, antiretroviral therapy; LNs, lymph nodes; cART, conventional antiretroviral therapy; Tfh, T follicular helper.
