# Supplementary material for: Blood CXCR3+ CD4 T Cells Are Enriched in Inducible Replication Competent HIV in Aviremic Antiretroviral Therapy-Treated Individuals
Source: Front Immunol. 2018 Feb 5;9:144. doi: 10.3389/fimmu.2018.00144 (PMC5807378; doi:10.3389/fimmu.2018.00144)

Supplemental Figure Legends

Supplemental Figure 1. Sorting strategy for chemokine receptor expressing blood memory CD4 T cell populations. Representative example of pseudo-colored flow cytometry plots of one aviremic ART-treated HIV-infected individual showing the sorting strategy of chemokine receptor expressing blood memory CD4 T-cell populations. Viable memory ($CD45RA^-$) CD4 T-cell sub-populations were sorted according to the expression of CXCR3, CXCR5, CCR4 and CCR6. Five chemokine receptor expressing CD4 T-cell populations were identified namely *i.e.* $CXCR3^+CXCR5^-$ (annotated as $CXCR3^+$), $CXCR3^-CXCR5^-CCR4^+CCR6^-$ (annotated as $CCR4^+$), $CXCR3^-CXCR5^-CCR4^+CCR6^+$ (annotated as $CCR4^+CCR6^+$), $CXCR3^-CXCR5^+$ (annotated as cTfh) and $CXCR3^+CXCR5^+$ CD4 T cells (annotated as Th1-like cTfh).

Supplemental Figure 2. CCR4 expression and CCR4, CCR6 co-expression on blood memory CD4 T-cell populations. Proportion of blood memory CD4 T-cell populations expressing either only CCR4 or co-expressing CCR4 and CCR6. Circles correspond to the percentage of $CCR4^+CCR6^+$ cells among the chemokine receptor expressing CD4 T cell population. Triangles correspond to the percentage of $CCR4^+$ cells among the chemokine receptor expressing CD4 T cell population. Red bars correspond to SEM**.** **"**X3" corresponds to blood CXCR3-expressing CD4 T cells; "X5" corresponds to blood CXCR5-expressing CD4 T cells; And $X3^+X5^+$ corresponds to blood $CXCR3^+CXCR5^+$ CD4 T cells. Statistical significance (*P* values) was obtained using One-way ANOVA (Kruskal-Wallis test) followed by paired t-test.

Supplemental Figure 3. Schematic representation of viral outgrowth assay. Sorted blood and lymph node memory ($CD45RA^-$) CD4 T-cell populations of ART-treated HIV-infected individuals with viral loads <50 HIV-1 RNA (copies/mL) (N=13) were cultured with allogeneic CD8-depleted blood mononuclear cells from HIV-1 negative subjects and HIV-1 replication (HIV-1 RNA copies/mL) was assessed at day 0 and 14 after anti-CD3 and anti-CD28 stimulation in culture supernatants by measuring HIV-1 RNA using RT-PCR.

Supplemental Figure 1

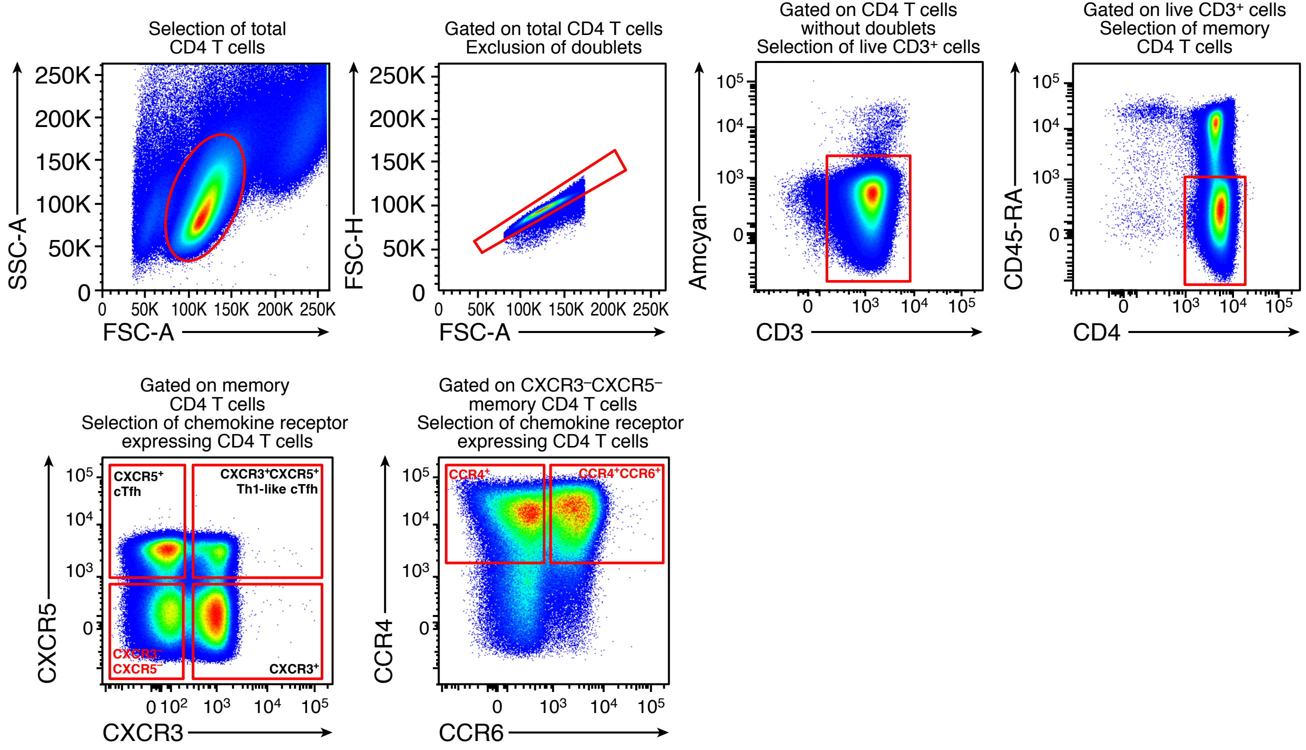

Supplemental Figure 2

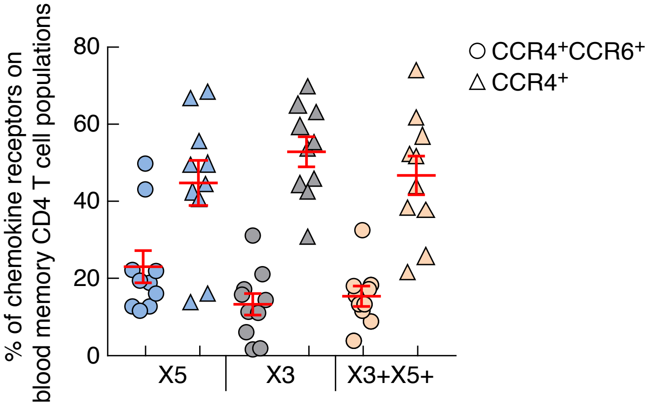

Supplemental Figure 3

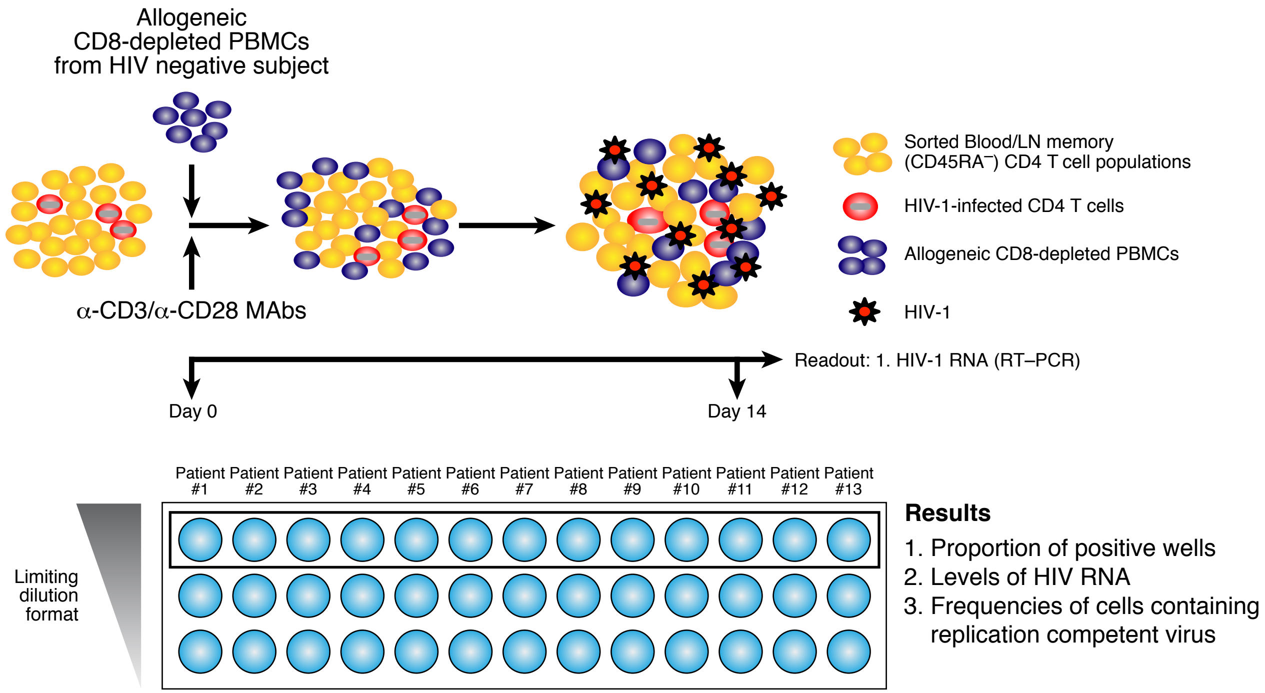

Supplement: Supplementary file 1 [file presentation_1.PDF]
