# Supplementary material for: Blood CXCR3+ CD4 T Cells Are Enriched in Inducible Replication Competent HIV in Aviremic Antiretroviral Therapy-Treated Individuals
Source: Front Immunol. 2018 Feb 5;9:144. doi: 10.3389/fimmu.2018.00144 (PMC5807378; doi:10.3389/fimmu.2018.00144)
Supplement: Supplementary file 2 [file table_1.PDF]

Supplemental Table 1. Estimation of contribution of blood memory chemokine receptor expressing CD4 T cell populations to the total pool of cells containing replication competent virus in blood

	**$CXCR3^+$**	**$CCR4^+$**	**$CCR4^+CCR6^+$**	**$CXCR5^+$**	**$CXCR3^+CXCR5^+$**
**Mean Freq. of CD4 T-cell populations in the cohort among blood memory CD4 T cells (%)**	22.7	26.4	8.5	13.7	8.9
**Mean Freq. of CD4 T-cell populations in the cohort among total blood CD4 T cells (%)***	14.7	17.2	5.5	8.9	5.8
**Number of CD4 T cells****	7.42E+08	8.64E+08	2.79E+08	4.49E+08	2.92E+08
**Freq. of cells containing replication competent virus per million (ELDA)**	12.9	4.3	5.0	4.1	2.2
**Number of cells containing replication competent virus*****	9.6E+03	3.7E+03	1.4E+03	1.8E+03	6.4E+02
**Contribution of each population to the reservoir in blood (%) *******	55.8	21.7	8.1	10.6	3.8

*Mean Freq. of CD4 T-cell populations in the cohort among total CD4 T cells = Mean Freq. of CD4 T-cell populations in the cohort among memory CD4 T cells x average Freq. of memory CD4 T cells (CD45RA-) among total CD4 T cells

**Number of CD4 T cells = Mean Freq. of CD4 T-cell population x number of CD4 T cells estimated by Ganusov *et al*. [33]

***Number of cells containing replication competent virus = Number of CD4 T cells x Freq. of cells containing replication competent virus (ELDA) / million

****Contribution of each population to the reservoir = Number of cells containing replication competent virus / sum of cells containing replication competent virus in the total compartment x 100
